# Rutin Is a Low Micromolar Inhibitor of SARS-CoV-2 Main Protease 3CLpro: Implications for Drug Design of Quercetin Analogs

**DOI:** 10.3390/biomedicines9040375

**Published:** 2021-04-02

**Authors:** Bruno Rizzuti, Fedora Grande, Filomena Conforti, Ana Jimenez-Alesanco, Laura Ceballos-Laita, David Ortega-Alarcon, Sonia Vega, Hugh T. Reyburn, Olga Abian, Adrian Velazquez-Campoy

**Affiliations:** 1 CNR-NANOTEC, Licryl-UOS Cosenza and CEMIF.Cal, Department of Physics, University of Calabria, 87036 Rende, Italy; 2 Institute for Biocomputation and Physics of Complex Systems (BIFI), Joint Units IQFR-CSIC-BIFI, and GBsC-CSIC-BIFI, University of Zaragoza, 50018 Zaragoza, Spain; ajimenez@bifi.es (A.J.-A.); ceballos.laita@gmail.com (L.C.-L.); dortega@bifi.es (D.O.-A.); svega@bifi.es (S.V.); 3 Department of Pharmacy, Health and Nutritional Sciences, University of Calabria, 87036 Rende, Italy; fedora.grande@unical.it (F.G.); filomena.conforti@unical.it (F.C.); 4 Departament of Biochemistry and Molecular and Cell Biology, University of Zaragoza, 50009 Zaragoza, Spain; 5 Institute for Health Research Aragón (IIS Aragon), 50009 Zaragoza, Spain; 6 Department of Immunology and Oncology, National Centre for Biotechnology (CNB), CSIC, 28049 Madrid, Spain; htreyburn@cnb.csic.es; 7 Aragon Health Sciences Institute (IACS), 50009 Zaragoza, Spain; 8 Biomedical Research Network Center in Hepatic and Digestive Diseases (CIBERehd), 28029 Madrid, Spain; 9 ARAID Foundation, Government of Aragon, 50018 Zaragoza, Spain

**Keywords:** rutin, quercetin, SARS-CoV-2, drug selection, enzyme inhibitors, antivirals, spectroscopy, molecular modeling

## Abstract

The pandemic, due to severe acute respiratory syndrome coronavirus 2 (SARS-CoV-2), has stimulated the search for antivirals to tackle COVID-19 infection. Molecules with known pharmacokinetics and already approved for human use have been demonstrated or predicted to be suitable to be used either directly or as a base for a scaffold-based drug design. Among these substances, quercetin is known to be a potent in vitro inhibitor of 3CLpro, the SARS-CoV-2 main protease. However, its low in vivo bioavailability calls for modifications to its molecular structure. In this work, this issue is addressed by using rutin, a natural flavonoid that is the most common glycosylated conjugate of quercetin, as a model. Combining experimental (spectroscopy and calorimetry) and simulation techniques (docking and molecular dynamics simulations), we demonstrate that the sugar adduct does not hamper rutin binding to 3CLpro, and the conjugated compound preserves a high potency (inhibition constant in the low micromolar range, *K*_i_ = 11 μM). Although showing a disruption of the pseudo-symmetry in the chemical structure, a larger steric volume and molecular weight, and a higher solubility compared to quercetin, rutin is able to associate in the active site of 3CLpro, interacting with the catalytic dyad (His41/Cys145). The overall results have implications in the drug-design of quercetin analogs, and possibly other antivirals, to target the catalytic site of the SARS-CoV-2 3CLpro.

## 1. Introduction

On January 2020, the World Health Organization (WHO) announced COVID-19 as a public health emergency of international concern and, on March 2020, declared severe acute respiratory syndrome coronavirus (SARS-CoV-2) a pandemic [1]. Since then, and to date, the new coronavirus has become a major global threat, with more than 100 million reported cases and over 2 million deaths worldwide. Scientists from all over the world are working hard in an extraordinary research mission to speed up the investigation for rapid diagnosis methods and for the development of vaccines and therapeutics to contain the spread of the infection. In less than one year, these efforts have led to the development and availability of safe and effective vaccines for COVID-19, which stimulate the immune system to produce specific antibodies able to block the spike viral protein and prevent the virus entry into the cells [2]. However, the need to cure patients already infected (or escaping from vaccination for various reasons) is a stringent necessity.

Although with little specific therapeutic indication for COVID-19, several drugs in clinical use for other disorders have been administered to patients infected with SARS-CoV-2 (for extensive reviews, see [3,4]). Many of these therapeutics belong to the class of antiviral agents, such as Remdesivir (a broad-spectrum pro-drug agent acting as a viral RNA-polymerase inhibitor), the combination Lopinavir/Ritonavir (HIV-1 protease inhibitors, which are together more efficient compared to the monotherapy of each drug), and Sofosbuvir (a nucleotide analogue targeting the hepatitis C virus (HCV) polymerase NS5B) in combination with Daclatasvir or Velpatasvir (both are potent HCV NS5A complex antagonists). However, and despite the huge number of research studies reported so far, the development of safe and effective drugs able to block the viral infection is still lacking and represents a major goal for the scientific world. Among all the possible chemical compounds, particular attention has been dedicated to molecules of natural origin [5,6,7,8], including extracts, single bioactive molecules, or entire classes of phytochemicals targeting SARS-CoV-2, and several of them are currently under investigation.

**Figure 1:**
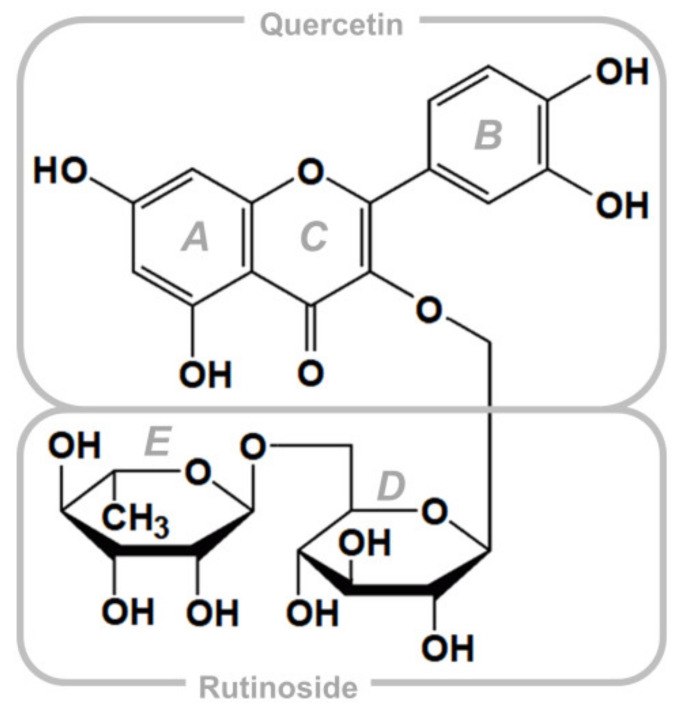
Chemical structure of rutin, with the quercetin scaffold (3,3′,4′,5,7-pentahydroxyflavone) and the rutinoside moiety (α-l-rhamnopyranosyl-(1→6)-β-d-glucopyranose). Rings (**A**–**E**) are labelled according to standard convention.

Rutin (quercetin-3-*O*-rutinose), shown in Figure 1, is a natural substance belonging to the flavonol class of flavonoids, widely distributed as secondary metabolites in several plants. Flavonoid compounds are made up by a phenylpropane-derivative and three acetyl-CoA (from malonyl-CoA) via the acetate-malonate biosynthesis pathway and cyclization via chalcones. For this reason, flavonoids possess (with only a few exceptions) an –OH group at positions C-5 and C-7. Relationships between the structure of flavonoids and their pharmacological activities have been proven so far; in particular, *C*-glycosidation enhances the effectiveness of antiviral and antibacterial activity [9]. Rutin is composed of one molecule of quercetin and the disaccharide rutinose. Quercetin 3-*O*-glycosides has a higher bioavailability (235%) compared to quercetin [10], and rutin is at least two times more soluble (130 mg/L) than its parent compound [11,12,13]. Quercetin and several of its conjugates have been approved by the U.S. Food and Drug Administration (FDA) for human use. Both quercetin and rutin are used as ingredients in numerous herbal remedies and have been extensively studied for their multiple pharmacological activities, including antiviral, antibacterial, and anti-inflammatory properties [9].

In a previous work [14], by using a combination of biophysical in vitro techniques, we had found that quercetin has the ability to inhibit the main protease of SARS-CoV-2, known as Mpro or 3CLpro (3C-like protease). 3CLpro is an excellent pharmacological target because it is highly conserved among the different members of the coronavirus family and no human host–cell proteases have been reported to show similar specificity. This protein is a crucial component of the viral replication machinery of SARS-CoV-2, as it is used to process the large polyproteins obtained by hijacking the host cell, to produce a number of key viral proteins that include 3CLpro itself. Quercetin was identified as the best hit in an experimental pipeline for drug screening that allowed us to identify many lead compounds against different protein targets in the recent years [15,16,17,18,19,20]. The screening library contains many drugs already approved for human administration, including natural compounds. Natural molecules bear remarkable biological qualities, and they are often found to be active against viruses [21,22].

The molecular scaffold of quercetin has a number of interesting physico-chemical properties that are attractive for a drug design endeavor. These features include low molecular mass, the presence of chemical groups that can be easily functionalized and, in the case of the use to target 3CLpro, considerable inhibitory activity (especially when scaled with respect to its molecular weight as an inhibitory efficiency index). Major shortcomings consist of a poor solubility and a very low bioavailability due to metabolic transformations after oral administration, which convert a high percentage of quercetin to glucuronide, methyl, and sulfate conjugates [23]. In general, these issues may be tackled by resorting to different strategies, such as employing controlled drug delivery system as nanocarriers circumventing solubility/trafficking/metabolic drawbacks or designing chemical modifications such as the introduction of chemical functional groups that can increase the solubility and thus the pharmacokinetic profile of the reference compound [24,25,26]. Furthermore, several chemical modifications have been proposed to improve the pharmacokinetics of quercetin in other cases [27,28]. Such modifications, however, open a number of key questions in the context of the use quercetin as antiviral against SARS-CoV-2. The two most important ones are perhaps the following: does the presence of a large adduct still guarantee a quercetin analog the ability to bind into the restricted pocket that constitutes the catalytic site of 3CLpro, and will the inhibitory effect of the parent compound still be retained?

To address these questions, we have used rutin as a test case to verify that the presence of the most commonly occurring sugar adduct, naturally attached to quercetin, allows the glycoside form to retain the key bioactive features of the aglycone lead compound. Although not constituting *per se* an optimization of quercetin towards a prescription drug, such validation constitutes an important proof-of-concept that could be considered a preliminary step to embark towards a more rational and challenging campaign of drug design. Our results, obtained by combining simulation and experimental techniques, show that rutin binds to the catalytic pocket of 3CLpro and, just like its parent compound quercetin, interacts with the dyad of protein residues responsible for the catalytic activity of the protease. More importantly, and although not being designed or optimized to this scope, rutin exerts a clear inhibitory activity with a relatively high potency against 3CLpro. These results are expected to encourage further investigations towards the development of quercetin-like antiviral compounds as a defense against coronavirus infections.

## 2. Materials and Methods

### 2.1. Protein Expression and Purification

SARS-CoV-2 3CLpro was expressed using a His-tagged construct in a pET22b plasmid transformed into BL21 (DE3) Gold *E. coli* strain. After initial cultures grown in LB/ampicillin (100 μg/mL) media at 37 °C overnight, 4 L of LB/ampicillin (100 μg/mL) were inoculated and incubated at 37 °C until reaching OD = 0.6 at a wavelength of 600 nm. Then, protein expression was induced with 1 mM isopropyl 1-thio-β-D-galactopyranoside (IPTG) at 18 °C for 5 h. Cells were harvested by centrifugation at 4 °C for 10 min at 10,000 rpm (Beckman Coulter Avanti J-26 XP Centrifuge, Barcelona, Spain) and then resuspended in lysis buffer (sodium phosphate 50 mM, pH 7, sodium chloride 500 mM). Cells were ruptured by sonication (Sonics Vibra-Cell Ultrasonic Liquid Processor, Newtown, CT) in ice, adding benzonase 20 U/mL (Merck-Millipore, Madrid, Spain) and lysozyme 0.5 mg/mL (Carbosynth, Compton, UK). Centrifugation at 4 °C for 30 min at 20,000 rpm, and filtration (0.45 μm-pore membrane) allowed removing cell debris from the extract. The protein was purified using affinity chromatography (ÄKTA FPLC System, GE Healthcare Life Sciences, Barcelona, Spain) using a cobalt HiTrap TALON column (GE-Healthcare Life Sciences), eluting by applying an imidazole 10–250 mM gradient. Purity was checked by SDS-PAGE (Figure S1), and pure protein fractions were dialyzed to remove imidazole and reach the protein storage condition (sodium phosphate 50 mM, pH 7, sodium chloride 150 mM). An extinction coefficient of 32890 M^−1^ cm^−1^ at 280 nm was employed for protein concentration quantification. Protein identity was assessed by mass spectrometry (LC-ESI-MS/MS).

### 2.2. Rutin Preparation

Rutin hydrate (purity ≥ 94%) in powder was purchased from Sigma-Aldrich (Milan, Italy). Solutions were prepared by dissolving the powder in pure DMSO at high rutin concentration (20 mM).

### 2.3. Circular Dichroism and Fluorescence Spectroscopy

Circular dichroism (CD) spectra were recorded in a Chirascan spectropolarimeter (Applied Photophysics, Leatherhead, UK) at 25 °C. Far-UV and near-UV spectrum were recorded at wavelengths between 190 and 250 nm in a 0.1-cm path-length cuvette, and between 250 and 310 nm in a 1 cm path-length cuvette, respectively, employing a protein concentration of 10 μM and a rutin concentration of 100 μΜ. Fluorescence measurements were performed in a Cary Eclipse fluorescence spectrophotometer (Agilent Technologies, Madrid, Spain), monitoring the intrinsic tryptophan fluorescence of the protein at 2 μM concentration. An excitation wavelength of 290 nm was used, with excitation and emission bandwidths of 5 nm, and recording fluorescence emission between 300 and 400 nm. All spectroscopic measurements were made in sodium phosphate 50 mM, pH 7, dimethyl sulfoxide (DMSO) 0.5%.

### 2.4. Proteolytic Activity Assay

In vitro catalytic activity of 3CLpro was monitored using a Förster resonance energy transfer (FRET) continuous assay with the substrate (Dabcyl)KTSAVLQSGFRKME(Edans)-NH2 (Biosyntan GmbH, Berlin, Germany) [29,30]. Briefly, the enzymatic reaction was initiated by adding substrate at 20 µM final concentration to the enzyme at 0.2 µM final concentration in a final volume of 100 µL. The reaction buffer was sodium phosphate 50 mM, pH 7, NaCl 150 mM, DMSO 2.5%. Fluorescence was measured in a FluoDia T70 microplate reader (Photon Technology International, Birmingham, NJ, USA) for 20 min (excitation wavelength, 380 nm; emission wavelength, 500 nm). Enzyme activity was quantified as the initial slope of the time evolution curve of the fluorescence signal. The Michaelis–Menten constant, *K*_m_, and the catalytic rate constant or turnover number, *k*_cat_, were estimated previously (*K*_m_ = 11 µM and *k*_cat_ = 0.040 s^−1^) [14].

### 2.5. Inhibition Assay

To assess the in vitro inhibition potency of rutin, the inhibition constant was estimated from the experimental inhibition curve. The inhibition curve was obtained by measuring the enzyme activity as a function of compound concentration: enzyme at 0.2 µM final concentration was incubated with rutin concentration from 0 to 120 µM, while maintaining constant the percentage of DMSO (2.5%), and the reaction was initiated by adding substrate at 20 µM final concentration [14]. The enzymatic activity was quantitated as the initial slope of the substrate fluorescence emission time curve and was plotted as a function of compound concentration. The slope ratio between the activity in the presence and absence of rutin provides the percentage of inhibition at a certain rutin concentration. Nonlinear regression analysis employing a simple inhibition model (considering inhibitor depletion due to enzyme binding) allowed us to estimate the apparent inhibition constant for rutin, according to Equation (1), by monitoring the substrate fluorescence emission as a function of time [14]:

(1) $$\begin{array}{c} \left[EI\right]=\frac{1}{2}\left({\left[I\right]}_{T}+{\left[E\right]}_{T}+K_{i}^{app}-\sqrt{{\left({\left[I\right]}_{T}+{\left[E\right]}_{T}+K_{i}^{app}\right)}^{2}-4{\left[E\right]}_{T}{\left[I\right]}_{T}}\right)\\ \left[I\right]={\left[I\right]}_{T}-\left[EI\right]=\frac{1}{2}\left({\left[I\right]}_{T}-{\left[E\right]}_{T}-K_{i}^{app}+\sqrt{{\left({\left[I\right]}_{T}+{\left[E\right]}_{T}+K_{i}^{app}\right)}^{2}-4{\left[E\right]}_{T}{\left[I\right]}_{T}}\right)\\ \frac{v\left(\left[I\right]\right)}{v\left(\left[I\right]=0\right)}=1-\frac{\left[EI\right]}{{\left[E\right]}_{T}}=\frac{1}{1+\frac{\left[I\right]}{K_{i}^{app}}} \end{array},$$

where [*EI*] is the concentration of the enzyme-inhibitor complex, [*E*]_*T*_ and [*I*]_*T*_ are the total concentrations of enzyme and inhibitor, $K_{i}^{app}$ is the apparent inhibition constant for the inhibitor (rutin), [*I*] is the concentration of free inhibitor, and *v* is the initial slope of the enzymatic activity trace at a certain (free) inhibitor concentration [*I*] (corresponding to a total inhibitor concentration [*I*]_*T*_). No approximation consisting of the free inhibitor concentration assumed equal to the total inhibitor concentration was made, thus having general validity for any total enzyme and inhibitor concentration and any value of the inhibition constant (even for tight binding inhibitors). If the inhibitor acts through a purely competitive mechanism, the previous equation can be substituted by Equation (2) [14]:

(2) $$\frac{v\left(\left[I\right]\right)}{v\left(\left[I\right]=0\right)}=\frac{1}{1+\frac{\left[I\right]}{K_{i}\left(1+\frac{\left[S\right]}{K_{m}}\right)}},$$

where *K*_i_ is the intrinsic (i.e., substrate concentration-independent) inhibition constant, *K*_m_ is the Michaelis–Menten constant for the enzyme–substrate interaction, and [*S*] is the substrate concentration. Because *K*_m_ and [*S*] are known, the intrinsic inhibition constant can be determined.

### 2.6. Isothermal Titration Calorimetry

Target engagement for rutin against 3CLpro was further assessed by isothermal titration calorimetry (ITC). Calorimetric titrations were performed using an Auto-iTC200 calorimeter (MicroCal, Malvern-Panalytical, Malvern, UK). Protein at 10 μM, located in the calorimetric cell, was titrated with rutin at 100 μM, performing experiments in two different buffers: Tris 50 mM, pH 7, and phosphate 50 mM, pH 7, with DMSO 1%. The experimental protocol consisted of a series of 19 injections of 2 μL each, using a stirring speed of 750 rpm, maintaining a spacing between injections of 150 s, and applying a reference power of 10 μcal/s. The association constant, *K*_a_, the binding enthalpy, Δ*H*, and the binding stoichiometry, *n*, (or percentage of active protein in the cell) were estimated through nonlinear least-squares regression analysis of the data, by using a model considering a single ligand binding site, implemented in Origin 7.0 (OriginLab, Northampton, MA, USA). The dissociation constant, *K*_d_, the binding Gibbs energy, Δ*G*, and the binding entropy, −*T*Δ*S*, were obtained from basic thermodynamic relationships. By performing titrations in buffers with different ionization enthalpies, the buffer-independent enthalpic, Δ*H*_0_, and entropic contributions, –*T*Δ*S*_0_, as well are the number of protons exchanged upon complex formation, *n_H_*, was estimated through linear regression of the observed enthalpy as a function of the ionization enthalpy of the buffer Δ*H*_buf_ (Δ*H* = Δ*H*_0_ + *n_H_* Δ*H*_buf_).

### 2.7. Molecular Docking

The simulation engine AutoDock Vina [31] and the modeling package AutoDock Tools [32] were used for docking experiments. The two reference crystallographic structures 6Y2E and 6Y2F [30] retrieved from the Protein Data Bank (PDB) were used, which contain 3CLpro in unliganded form and complexed with an α-ketoamide inhibitor bound in the catalytic protein site, respectively. Three missing residues in the latter were reconstructed in silico, and the ligand and water molecules were not considered in the docking. The structure of rutin was taken from PDB entry 1RY8 [33], and it was improved by performing an energy minimization using UCSF Chimera [34] and with the addition of the hydrogens. A blind docking search with very high exhaustiveness [35] was performed on a volume including the whole protein. The number of poses obtained for the two protein targets were reduced by performing a selection based on the binding energy (affinity within 1.5 kcal/mol from the best docking pose) and structure similarity (atomic root mean square deviations RMSD < 2 Å).

### 2.8. Molecular Dynamics

The 3CLpro-rutin complexes obtained after molecular docking were refined in molecular dynamics (MD) simulations performed using the GROMACS suite [36]. The complexes were solvated in a rhombic dodecahedral box with a distance of 10 Å from the closest edge, resulting in more than 20,000 water molecules added, and four Na^+^ counterions were included to neutralize the system. The force field Amber ff99SB-ILDN [37] was used for the protein, GAFF [38] for the ligand, and the TIP3P model for water [39]. Production runs were performed for 10 ns in the isobaric-isothermal ensemble, after a standard preparation routine including energy minimization, annealing, and equilibration [40]. Other simulation conditions, which include the parameters for the thermostat/barostat, the modeling of electrostatic and non-electrostatic interactions, and the use of constraints, were as previously described [41,42]. In the subsequent analysis, distances and root mean square fluctuations (RMSFs) were calculated after eliminating the protein rototranslation by a least-squares fit with respect to the C^α^ atoms. Distances from His41 and Cys145 were calculated by considering the geometric center of, respectively, the five non-hydrogen atoms in the imidazole ring and the three C^α^-C^β^-S^γ^ atoms in the side chain of these two residues.

## 3. Results

### 3.1. Rutin Binds and Inhibits the Main Protease 3CLpro

**Figure 2:**
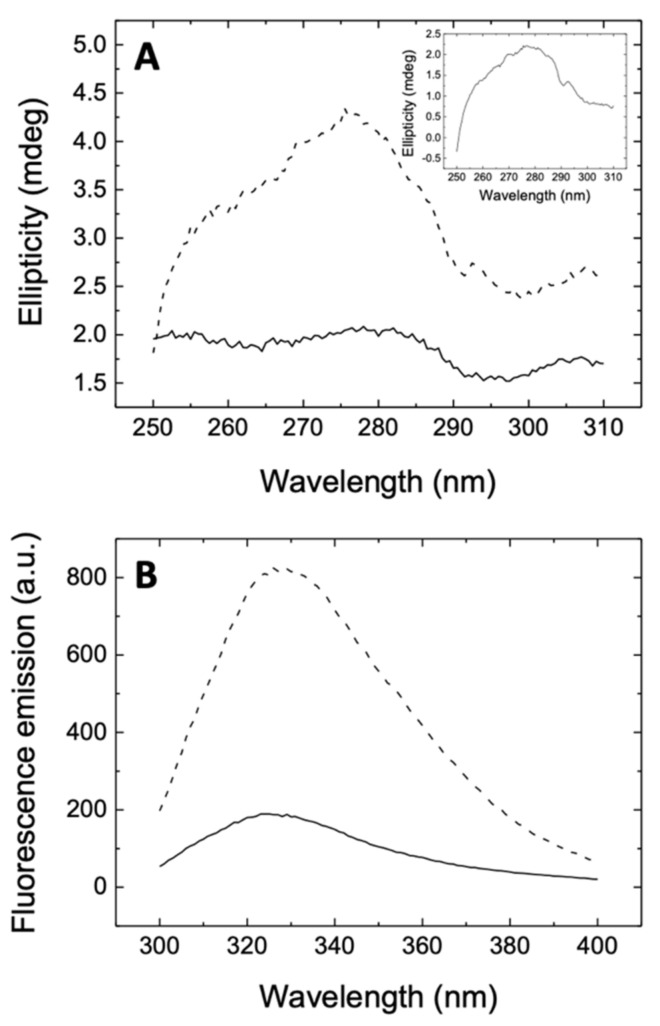
(**A**) Near-UV CD spectrum of the 3CLpro-rutin complex (continuous line) and addition of individual spectra of 3CLpro and rutin (dashed line), recorded at 10 μM protein concentration and 100 μM rutin concentration. Inset: Near-UV spectrum of 3CLpro. The non-equivalence of the spectrum of the complex and the addition of the spectra of free species is the result of the interaction. (**B**) Fluorescence emission spectrum (in arbitrary units, a.u.) of 3CLpro-rutin complex (continuous line) and addition of individual spectra of 3CLpro and rutin (dashed line), recorded at 2 μM protein concentration and 100 μM rutin concentration. Rutin showed negligible fluorescence emission; therefore, the dashed line also corresponds to the emission spectrum of 3CLpro.

The far-UV CD spectrum is informative about the conformational state of a protein, providing quantitative information on its secondary structural motifs. However, in cases where protein–ligand interaction does not result in sufficiently large conformational changes, the spectrum may not reflect that interaction, as observed in 3CLpro interacting with rutin (Figure S2). On the other hand, near-UV CD is more difficult to interpret on a structural basis, but it is more sensitive to subtle changes in the tertiary structure and the environment of aromatic residues. Therefore, near-UV spectra were determined in order to provide direct evidence of 3CLpro-rutin interaction, as shown in Figure 2A. In addition, the presence of rutin strongly affected the intrinsic tryptophan fluorescence emission, as shown in Figure 2B. Because the fluorescence of rutin is negligible, the addition of both individual fluorescence spectra was almost identical to that of the free protein. The strong quenching effect of rutin was indicative of the interaction with the protein. Rutin modified the spectroscopic properties of 3CLpro, which in turn demonstrates that the ligand (i) binds to its pharmacological target, and (ii) has the ability to alter the tertiary structure of the protein and/or the environment of aromatic protein side chains to a significant extent. The spectral distortions caused by rutin on 3CLpro are similar to those previously observed for quercetin [14], and may be ascribed in both cases to a destabilization effect of the ligand on 3CLpro.

**Figure 3:**
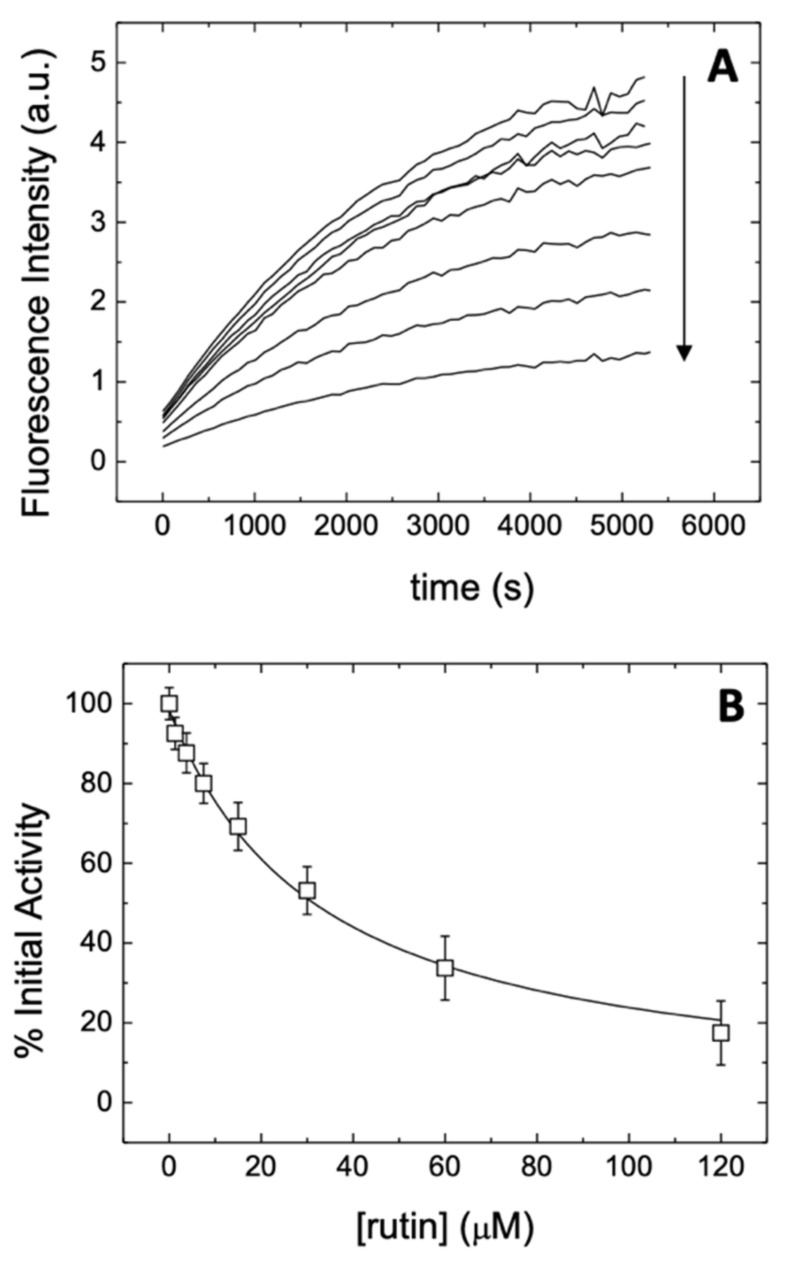
(**A**) Fluorescence emission (in arbitrary units, a.u.) of the substrate as a function of time at varying concentration of rutin. The concentration of the substrate and 3CLpro were fixed at 20 and 2 μM, respectively, while the concentration of rutin was varied from 0 to 120 μM following a two-fold serial dilution. The arrow indicates the increase in rutin concentration; (**B**) Experimental inhibition curve for rutin (initial slope as a function of total rutin concentration). Nonlinear least-squares regression data analysis (continuous line) according to Equations (1) and (2) provided an apparent inhibition constant *K*_i_^app^ of 31 μM, and an intrinsic inhibition constant *K*_i_ of 11 μM.

The ability of rutin to hamper the enzymatic activity of 3CLpro was assessed by observing the inhibitory action as a function of the ligand concentration, as shown in Figure 3. The intensity of the fluorescence signal could be monitored as a function of time (Figure 3A), by fixing the concentration of both the substrate and the protein. The curves show a more rapid and almost linear increase in the first few minutes, and afterwards the emission continues to grow more gradually up to about 1.5 h. The increase in fluorescence emission reflects the proteolytic activity of 3CLpro as a reduction in the FRET effect due to the cleavage of the substrate and the concomitant spatial separation of FRET donor and acceptor. The initial slope provides a direct quantification of the proteolytic activity of 3CLpro. Increasing the concentration of rutin (tested up to 120 μM) resulted in a reduction of the initial slope of the fluorescence trace. These observations indicate a decrease in the enzymatic activity of the main protease 3CLpro as a consequence of the presence of rutin, showing a concentration-dependent action. The effect is qualitatively very similar to the one previously observed for quercetin [14], indicating that conjugation with the glycoside moiety does not substantially hamper the inhibitory effect of the flavonoid molecular scaffold. This experimental finding is a first but already strong indication that the sugar region of rutin has only an auxiliary role in the binding to 3CLpro, encouraging further exploration of the potential of quercetin analogs to target this protein.

A more direct indication of the actual potency of rutin for inhibiting the enzymatic activity of 3CLpro could be obtained by determining the initial slope of each of the fluorescence curves, to evaluate the activity as a function of the ligand concentration (Figure 3B). The inhibition curve obtained showed in a straightforward way the dose-dependent effect of the presence of this compound on the functionality of 3CLpro. More importantly, the data could be fit by using a nonlinear regression model based on a simple inhibition process (see the section Materials and Methods). The analysis yielded an apparent inhibition constant *K*_i_^app^ = 31 μM, which can be compared with the value of 21 μM previously obtained for the parent compound quercetin [14]. Under the hypothesis that the binding of rutin takes place into a single protein site (which is later validated in the following sections), the apparent inhibition constant can be used to evaluate the so-called intrinsic inhibition constant (see again the section Materials and Methods). By using this model, which also accounts explicitly for the substrate concentration and the occurrence of a competitive inhibition, we obtained for rutin an intrinsic inhibition constant *K*_i_ = 11 μM. This value can be again directly compared with the one obtained for quercetin, *K*_i_ = 7.4 μM [14], and is comparable with those reported for known inhibitors described in the literature for the previous coronavirus species SARS-CoV [43].

**Figure 4:**
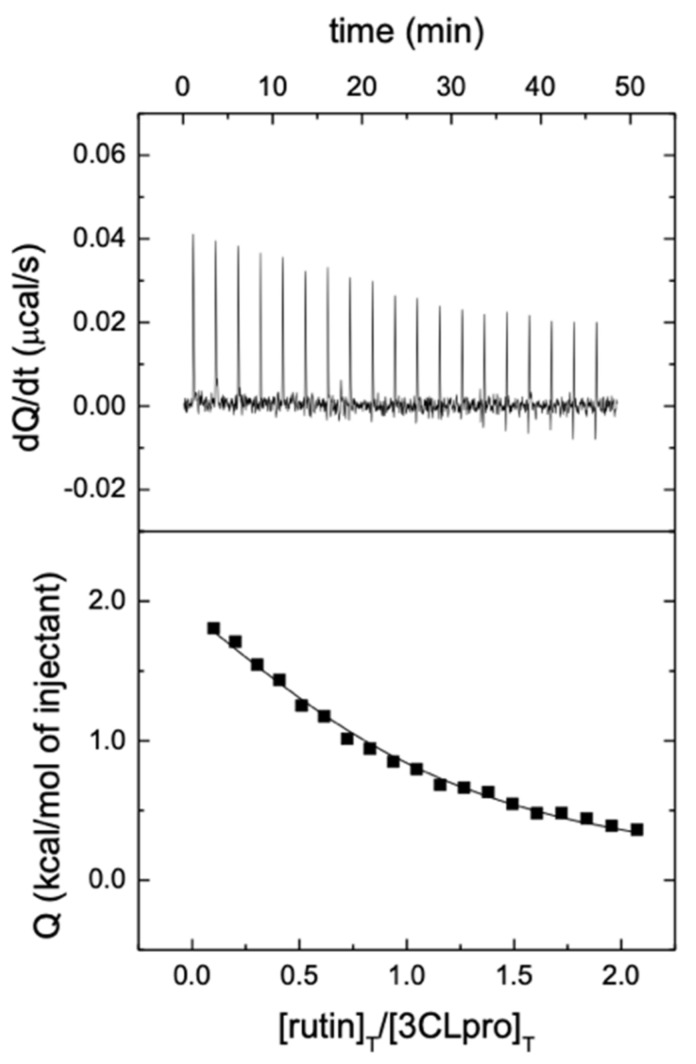
Interaction of rutin with 3CLpro assessed by isothermal titration calorimetry at 25 °C in Tris buffer, pH 7. The upper plot shows the thermogram (thermal power required to maintain a null temperature difference between sample and reference cells as a function of time) and the lower plot shows the binding isotherm (ligand-normalized heat effect per injection as a function of the molar ratio, the quotient between the ligand and protein concentrations in the cell). The fitting curve corresponds to the single ligand binding site model (continuous line). According to the data analysis, rutin interacts with 3CLpro with unfavorable enthalpic contribution (Δ*H* = 3.4 kcal/mol) and favorable entropic contribution (−*T*Δ*S* = −10.4 kcal/mol) to the Gibbs energy of binding (Δ*G* = −7.0 kcal/mol), corresponding to a dissociation constant *K*_d_ of 6.9 μM. The fraction of active (or binding-competent) protein is 85% (*n* = 0.85).

Another direct piece of evidence for the interaction of rutin with 3CLpro, as well as a quantitative determination of the dissociation constant (equivalent to the intrinsic inhibition constant), was obtained by ITC, as shown in Figure 4. ITC is the gold standard for binding affinity determination, and it also allows the determination of the binding enthalpy and stoichiometry. According to the results, the interaction of rutin with 3CLpro in Tr. is buffer is characterized by a dissociation constant *K*_d_ = 6.9 μM and an interaction enthalpy Δ*H* = 3.4 kcal/mol, whereas the interaction in phosphate is characterized by a dissociation constant *K*_d_ = 6.7 μM and an interaction enthalpy Δ*H* = −5.1 kcal/mol. From these data, an average dissociation constant *K*_d_ = 6.8 μM, a buffer-independent interaction enthalpy Δ*H*_0_ = -5.8 kcal/mol, and a net number of exchanged protons *n_H_* = 0.8 (protonation of complex upon binding) could be estimated. From that, a favorable albeit small buffer-independent entropic contribution (−*T*Δ*S*_0_ = −1.3 kcal/mol), could be calculated. Therefore, the interaction is entropically and enthalpically favorable, but dominated by enthalpic interactions, with a Gibbs energy of binding Δ*G* = −7.0 kcal/mol. The dissociation constant compares fairly well with the intrinsic inhibition constant, *K*_i_, previously measured. Compared to quercetin, there is a similar character for the binding of rutin, but the binding is driven by enthalpic effects, which may reflect a stabilization of the quercetin scaffold in the binding site due to (or in combination with) additional interactions of the glycoside moiety.

To summarize our findings on the effect of rutin towards the main protease 3CLpro from SARS-CoV2, the experimental evidence demonstrates rutin target engagement and point out a clear inhibitory action on the protein catalytic activity. The inhibition constant of rutin, although lower than that observed for its sugar-depleted parent compound, is still in the low micromolar range, indicating a sufficiently strong inhibition potency also for this ligand.

### 3.2. 3CLpro-Rsutin Interaction Takes Place in the Catalytic Site

**Figure 5:**
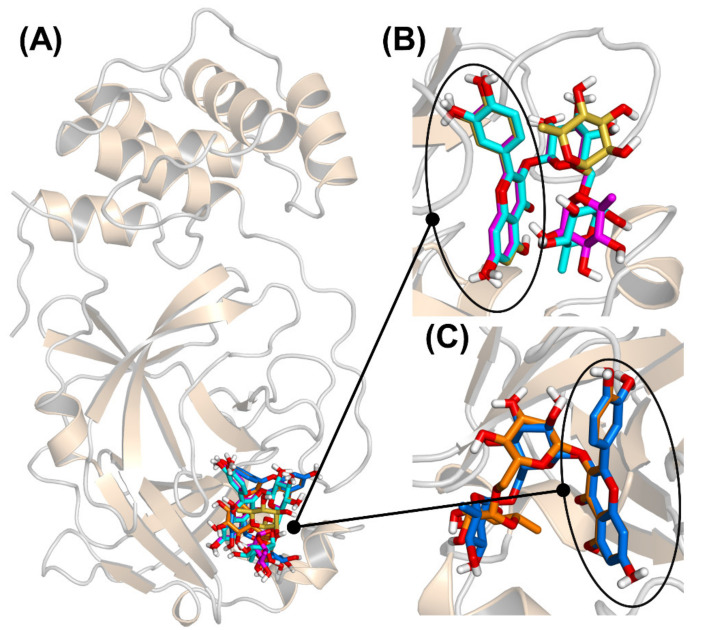
Docking poses of rutin in the active site of the main protease 3CLpro, shown at different rotation angles in the three panels. (**A**) Ribbon representation of the protein, with the most favorable docking poses superimposed; (**B**) Cluster of three docking poses (cyan, magenta, and yellow), with the quercetin moieties (circled) almost coincident, and the rutinose regions pointing outwards; (**C**) Cluster of two docking poses (blue and orange); the quercetin moieties are still almost coincident, and the rutinose regions extend on the other side with respect to the other cluster (panel B).

To investigate the binding of rutin at the catalytic site of 3CLpro, molecular docking was initially employed. Two reference crystallographic structures [30] were used, which contain 3CLpro either in unliganded form or complexed with an inhibitor in the catalytic protein site. After a blind search performed with a very high exhaustiveness on the whole surface of both (ligand-free) protein structures, the most favorable binding modes obtained with these two docking hosts were analyzed and compared. The simulation results are shown in Figure 5. The docking poses accumulated in the 3CLpro catalytic site (Figure 5A) in terms of both the number of different binding modes and the most favorable affinity scores. After clustering the poses to account for similarity in their structures, the most favorable ones were found to belong to two sole groups (see Figure 5B,C). These two groups had in common the fact that the quercetin moiety was clearly anchored to the 3CLpro binding site, whereas rutinose interacted only through a fraction of its chemical groups with the protein pocket. Furthermore, in each group, the different binding modes had their quercetin scaffold virtually superimposed, whereas the glycoside regions were more disordered. These results suggest that the flavonoid moiety of rutin is fundamental to bind 3CLpro, whereas the rest of the molecule forms less specific interactions with the protein.

The binding energies calculated in our docking experiments ranged from –7.5 to –9.0 kcal/mol. These values could be considered upper bounds or even overestimations of the actual affinity, which, according to the intrinsic inhibition constant would be –6.8 kcal/mol (and could be compared to −7.0 kcal/mol for quercetin, as calculated according to its intrinsic inhibition constant). In fact, docking simulations account only implicitly for the presence of solvent, whereas, in reality, rutinose (and especially its hydroxyl groups) could be expected to interact preferentially with water molecules at the protein surface. We verified that redocking of the sole quercetin moiety of rutin (with the whole disaccharide moiety substituted in position C-3 with a single methyl group), in the same location previously found in the docking experiments and without performing any search, yielded a binding score of −6.8 kcal/mol. This result confirms that the quercetin region of rutin is essentially responsible for the binding of the whole molecule, whereas the glycoside region plays only a margin role. All these findings agree with the observation that the parent compound quercetin has a binding free energy of about −7 kcal/mol, as we had previously measured by both isothermal titration calorimetry (ITC) experiments and docking simulations performed with the same protocol here used for rutin [14]. It is unlikely that the affinity of quercetin could be drastically improved through the addition of a bulky and largely polar chemical adduct with little specificity towards the protein surface.

**Figure 6:**
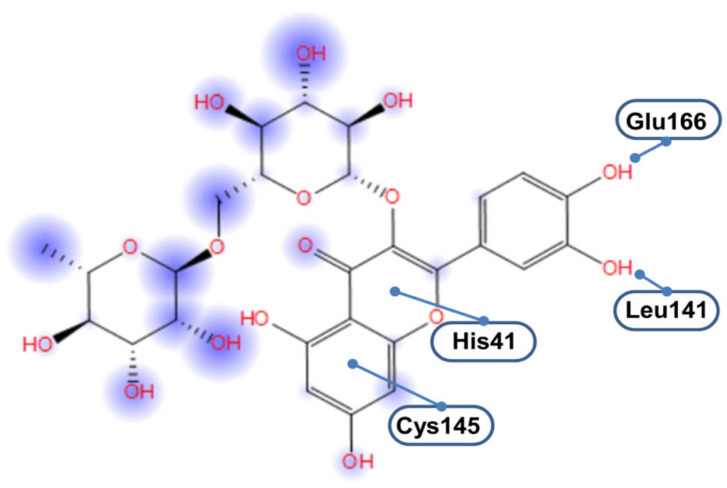
Schematic representation of the interactions of rutin within the active site of 3CLpro. Circular halos around rutin atoms are proportional to solvent exposure.

An important point is the description of the molecular interactions that guarantee the binding of rutin to 3CLpro, and especially of its quercetin scaffold. In particular, it is worth clarifying whether the interaction is mediated by the two protein residues forming the catalytic dyad, His41, and Cys145. In principle, a direct involvement of these two residues in the binding is not entirely obvious because the overall active site of 3CLpro has a relatively extended shape that includes 24 amino acids and a surface area of 235 Å^2^ (as calculated by using a solvent probe with radius 1.4 Å through the CASTp algorithm [44]). Nevertheless, as illustrated in Figure 6, the double ring *A*/*C* of the quercetin moiety forms direct interactions with the two residues His41/Cys145. The rest of the scaffold (i.e., quercetin ring *B*) has a higher conformational freedom, and can form hydrogen bonds as a donor with either the backbone oxygen of Leu141 or the carboxylate group in the side chain of Glu166. The disaccharide region of rutin, in sharp contrast with the flavonoid scaffold, appears to be much more exposed to the solvent.

### 3.3. Binding of Quercetin Scaffold Is Not Hampered by a Bulky Adduct

Molecular docking simulations are useful to perform a blind exploration of a protein, for predicting the binding of a ligand to its host in a reasonable amount of elapsed real time. However, the docking technique has several limitations [45], which notably include the following ones: (i) the protein receptor is considered rigid to simplify the search; (ii) the solvent is accounted for in an implicit way only; and, more generally, (iii) no information can be determined on the behavior of the solvated complex as a function of time. The first point is important because it may preclude a fine accommodation of the ligand in the binding site. The second point is also particularly relevant in our specific case because of the more hydrophilic nature of the sugar region of rutin compared to the quercetin scaffold. To overcome these issues, all-atoms MD simulations in explicit solvent were performed starting from the complex structures predicted by the docking technique. The simulations were carried out for 10 ns, which is a time scale sufficient to explore the local dynamics of the ligand in the binding pocket. Large scale modifications of 3CLpro as a consequence of the binding of rutin would require a much longer time scale and lie in the realm of state-of-the-art computations [46,47].

**Figure 7:**
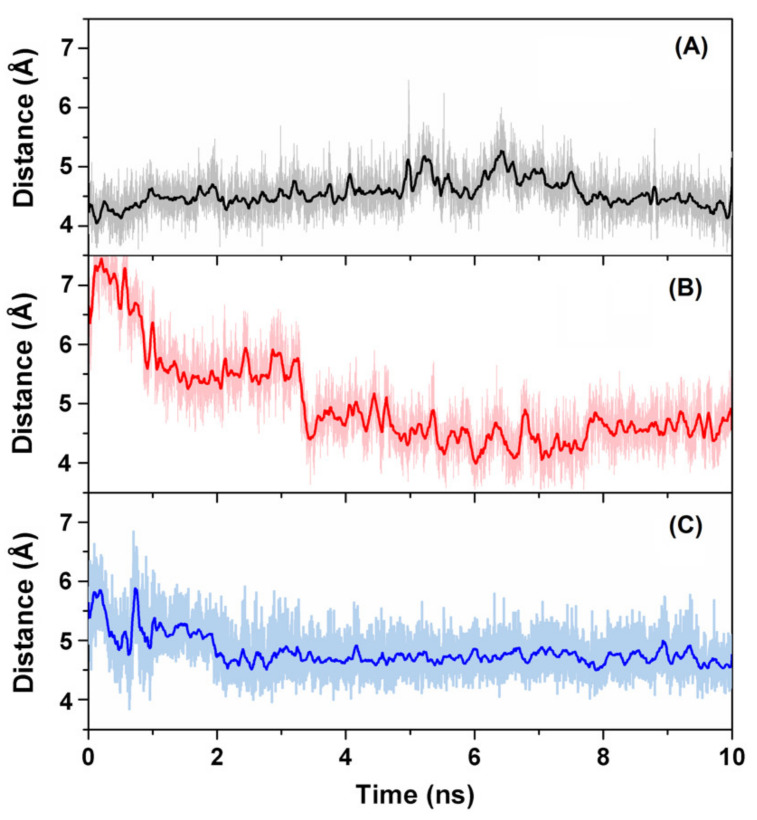
Distance between the double ring in the quercetin moiety of rutin and the catalytic residues His41/Cys145 of 3CLpro in MD simulation. (**A**) Distance with respect to the imidazole ring of His41, for the most favorable docking pose; (**B**) Separation from the side chain ring of His41, starting from a docking pose sub-optimally accommodated in the binding pocket; (**C**) Separation from the side chain of Cys145 (same simulation run as in B).

A direct way to confirm the interaction of rutin with the 3CLpro catalytic dyad His41/Cys145 is to estimate the distance between these two protein residues and the non-glycoside moiety of the ligand, as reported in Figure 7. In the case of the most favorable docking poses (Figure 7A), interaction with the side chain of His41 was already stable at the start of the simulation and remained so during the whole MD run, with an equilibrium distance of 4.5 ± 0.3 Å. This value can be compared with the typical ring-ring distance for π–π interactions, which is <4 Å in the most favorable cases but may increase up to 4.5 Å in many practical situations [48,49]. In our case, deviations from the ideal case could be easily ascribed to the dynamics of the protein–ligand complex. We also verified that, in a simulation run starting from a less favorable docking pose (affinity score -7.5 kcal/mol) within the catalytic pocket, the ligand took about 3.5 ns for the heterocyclic ring *C* to reach the equilibrium distance (again at 4.5 ± 0.3 Å, see Figure 7B) with respect to the ring of His41. In the same simulation, 2 ns were necessary for rutin to accommodate its aromatic ring *A* with respect to the side chain of Cys145 (equilibrium distance thereafter was 4.7 ± 0.3 Å, as visible in Figure 7C).

**Figure 8:**
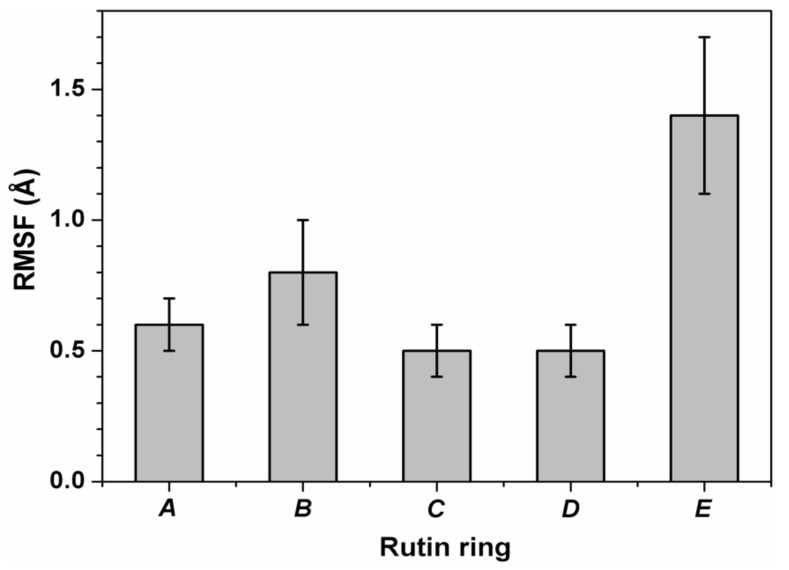
Root mean square fluctuations (RMSF) of the inner motion for the five rings of rutin. Rings *A* and *C* together form the connected double ring of the quercetin moiety, and rings *D* and *E* belong to the disaccharide rutinose (see also Figure 1). Values are calculated for the six non-hydrogen atoms forming each ring. Error bars indicate the uncertainties in terms of standard deviations.

The inner dynamics of rutin were also assessed by calculating the atomic fluctuations for each of the rings in its molecular structure, after removing non-internal motions of the ligand anchored to 3CLpro due to the diffusive motion of the protein in the solvent. The results reported in Figure 8 show that fluctuations were smaller (RMSF < 1 Å) for the quercetin region of rutin, and relatively larger (RMSF > 1.5 Å) for the outmost ring in the sugar region (i.e., ring *E*). Movements in the latter regions led to fluctuations >2 Å for the hydroxyl O atoms, preventing the formation of stable hydrogen bonds with the protein. These findings further support the notion that the quercetin scaffold plays a prevalent role in anchoring the whole ligand to the protein, whereas the sugar region is mostly disordered. It is also interesting to note that, although the dynamics of the ligand tends to improve its accommodation within the protein site compared to the starting position, the binding affinity of rutin at the end of the MD simulations did not increase compared to the value originally estimated by applying the sole molecular docking techniques. In fact, re-docking experiments on the rutin-3CLpro complexes obtained at the end of the MD runs, performed by using the same scoring function without any search for the ligand position, showed a binding energy of −7.5 kcal/mol. As already verified in the case of molecular docking, the quercetin moiety of rutin gave the major contribution to this value, whereas the contribution of the sugar region was marginal.

## 4. Discussion

Pharmacological research to fight against SARS-CoV-2 is hectic at present, and large efforts are devoted to the search for antiviral agents [7]. A number of potential candidates have already been proposed [50], based on experiments and computational predictions, and many collaborations are active to find new ones [51]. However, emergence of an increasing number of mutations in SARS-CoV-2 and the necessity to prevent drug resistance calls for further attempts along this direction. Among the strategies to block SARS-CoV-2 infection, inhibiting the main protease 3CLpro is perhaps the most appealing. In fact, this protein is vital in the replication process of all coronaviruses. 3CLpro shows a high degree of homology among different members of this family, as well as among different strains of SARS-CoV-2. Furthermore, amino acid residues in the active site are highly conserved. In addition, 3CLpro shows little homology with host–cell proteases, thus minimizing potential side-effects. Although indirect ways of inhibiting this protein could be envisioned (e.g., shifting the monomer–homodimer equilibrium toward the inactive monomeric state, or exploiting the existence of binding sites with allosteric effects), the most direct way is to tackle the catalytic binding site, and in particular the dyad of residues His41/Cys145. A number of inhibitors were found to have the ability to inhibit 3CLpro, including covalent ones. However, most of these molecules are expected to have severe side effects or other limitations that will preclude their direct use as an antiviral drug.

Among the compounds showing antiviral effects against 3CLpro, we previously demonstrated that a very active one is quercetin [14], which appears to be very interesting also because of its known pharmacokinetic properties and high tolerability. Large availability, low cost, and the absence of encumbering patents are also quite attractive features to use quercetin as a scaffold for drug design [27]. In order to prove the potential benefit in using quercetin as a starting point for such an endeavor, and before moving to more complex attempts (e.g., a modification of the central core of the structure to obtain active analogs through a scaffold hopping process), some modifications should be tested. First, quercetin has a low solubility [13]; therefore, it should be assessed whether the presence of a more hydrophilic chemical adduct still allows this compound to bind and inhibit 3CLpro. Second, quercetin has a low molecular mass (302 Da), which is very close to the threshold conventionally used to classify a compound as a chemical fragment (<300 Da), thus modifications that increase its mass and steric hindrance need to be considered. Finally, we previously noted that the pseudo-symmetry in the structure of quercetin could play a role in binding to 3CLpro [14]; therefore, the effects of the presence of a large symmetry-breaking adduct that may modify this feature should be probed.

To address all these points, we have investigated both in vitro and in silico rutin, a quercetin analog that includes a two-ring sugar moiety conjugated with the core scaffold, and with a larger solubility. Rutin is also a well-known natural product, extensively used for various pharmaceutical properties in hundreds of registered preparations [52]. It has also been proved to have no cytotoxic effects towards different healthy human cell lines, including cultured normal cells (at concentrations up to 300 µM for 24 h), and in a variety of other cases such as for human umbilical vein endothelial (HUVE) cells, lung embryonic fibroblasts (TIG-1), and mammary fibroblasts [53,54].

The combination of our experimental and computational results points out that rutin binds to 3CLpro. This finding is demonstrated by alterations in the near-UV CD and fluorescence emission spectra, which demonstrate an engagement of the pharmacological target that produces modification in the structure of the protein. The simulation has difficulties to model these modifications due to the size of the (solvated) protein and, more importantly, due to the large timescale that needs to be probed to observe them. Therefore, a twofold computational approach was pursued. In a first step, molecular docking was employed to prove the binding of rutin in the catalytic site of 3CLpro, in blind experiments carried out considering the whole protein structure. The results showed that the protein active site, and more specifically the catalytic dyad His41/Cys145, provides an anchoring for the binding of rutin. Subsequently, MD simulation was used to refine the accommodation of the rutin, and to investigate the dynamics of the ligand. Our findings confirmed that the quercetin scaffold gives the main contribution, both from a structural and energetic point of view, whereas the rutinose moiety remains in contact with the solvent and plays a secondary role in the association. In particular, our simulation techniques provided details on how the double-ring structure of quercetin is the key binding interface with the His41/Cys145 dyad.

The resulting effect of the association of rutin on the active site of 3CLpro is an inhibitory action on 3CLpro catalytic activity that was clearly visible in our fluorescence results based on the Förster resonance energy transfer (FRET). This technique provides a measure of the hydrolytic activity of 3CLpro on a substrate, and it is therefore useful to estimate the degree of activity for small molecules capable to block the enzymatic activity of the protein. The inhibition constants obtained for rutin were, respectively, *K*_i_^app^ = 31 μM (apparent) and *K*_i_ = 11 μM (intrinsic). These values are slightly less favorable compared to those previously obtained for quercetin [14], 21 and 7.4 μM, respectively, a result that is particularly encouraging for the fact that rutin was not selected with the aim to improve the molecular properties of quercetin. The corresponding concentration of rutin necessary for a 50% inhibitory effect (IC_50_) can be readily estimated, and it has a value of IC_50_ = 32 μM, although it is important to note that this parameter may not be an appropriate inhibition potency index to measure in vitro inhibition because it is an assay-dependent value that would be different if another substrate or enzyme concentration were employed.

The inhibition constant found for rutin still compares well with the values obtained for other inhibitors specifically designed to bind 3CLpro. This constitutes an encouraging step to further explore the possibility of using other quercetin analogs to block the enzymatic activity of 3CLpro, including perhaps more radical alterations of the starting molecular scaffold. According to the inhibition constant and its molecular mass (610 Da), rutin shows a binding efficiency index (BEI = p*K*_i_/MW) of 8.1, compared to 16.9 for quercetin. This reduction in binding efficiency reflects the fact that the larger molecular mass does not result in more or significantly stronger interactions, since the glycoside moiety of rutin hardly interacts with 3CLpro. However, we must consider that the overall effect of a given inhibitor will be a combination of pharmacodynamic and pharmacokinetic properties; thus, a slightly lower inhibition potency for rutin might be favorably counterbalanced by its much better solubility and bioavailability.

The use rutin and other quercetin analogs to inhibit 3CLpro had already been proposed by several studies in the vast literature on SARS-CoV-2, together with a large number of other natural compounds. To the best of our knowledge, and with the notable exception of our previous work on quercetin [14], former predictions were based solely on computational analyses. They included molecular docking [55,56,57], with additional insights from both classical [58,59] and more advanced MD methods [60], or machine learning approaches [57]. In some cases, the compounds identified were further examined [56,61] in terms of their quantitative structure-activity relationship (QSAR) and expected pharmacokinetics properties including absorption, distribution, metabolism, excretion, and toxicity (ADMET). Our study differs from the others in a number of key aspects. Most significantly, we obtained the first experimental evidence of the inhibitory action of rutin towards 3CLpro. We also provided an estimation of the intrinsic inhibition constant, *K*_i_, suggesting that previous predictions were often quite inaccurate (in some cases by several order of magnitude [61]). We further demonstrated that rutin acts directly on the dyad of protein residues exerting the catalytic activity of 3CLpro, in agreement with most predictions and at variance with the hypothesis of an indirect, allosteric action [62]. More generally, we proved the importance to couple MD simulations to overcome limitations of the docking technique.

Comparison with some of the previous computational works, on the other hand, provides a number of interesting suggestions on how to further explore the potential of the quercetin scaffold to develop antiviral compounds against SARS-CoV2 main protease. Of special interest is the agreement on the potential use of glycosylated flavonoids, such as quercetin-3-*O*-rhamnoside [58], quercetin-3,5-digalactoside and quercetin-3,5-diglucoside [56]. In a more general context, this suggests that the use of quercetin and its analogs to target SARS-CoV-2 can benefits of the results already obtained for SARS-CoV and MERS-CoV, the viruses responsible for the previous coronavirus outbreak in 2002 and 2012, respectively, and which share a high sequence identity (>95%) for the main protease 3CLpro. Experiments had shown that quercetin had the ability to inhibit 3CLpro of both SARS-CoV [63] and MERS-CoV [22], and quercetin-3-β-galactoside and other synthetic derivatives were also active [64]. Thus, our findings add rutin to the list of quercetin-derived compounds whose antiviral properties are demonstrated in vitro against the coronavirus family.

## 5. Conclusions

The identification and design of antivirals compounds against SARS-CoV-2 is of utmost importance as one of the main ways to reduce the impact of COVID-19 infection on public health. In particular, inhibition of the catalytic activity of the main protease 3CLpro (or Mpro) is one of the best pharmacological strategies to block the viral replication in affected patients. High similarity of the active site among different variants of SARS-CoV-2, as well as other coronaviruses, makes this approach particularly intriguing also to reduce the incidence of present and possibly future infections due to other related viral strains. After the previously reported identification of quercetin as an excellent in vitro inhibitor of 3CLpro, herein we have shown that its glycosylated conjugate rutin is also effective to this scope, and with a comparable potency. Determining the antiviral effect in vivo on human cells of this compound, and possibly of its derivatives, will give a concrete answer to the possibility of the direct use as a pharmaceutical. In the meanwhile, this adds one more natural product to the list of molecules that are potentially active against SARS-CoV-2, which is of further interest due to the high tolerability of many of these compounds for human use. More importantly, our findings suggest that there is a large amount of room for the possibility of improving the flavonoid scaffold of quercetin to design more effective analogues. The molecular features of rutin/quercetin, including the presence of many hydroxyl groups that can be readily functionalized, offer a variety of possibility for future improvements.

## Data Availability

The data presented in this study are available from the corresponding authors upon reasonable request.

## Supplementary Materials

The following are available online at https://www.mdpi.com/article/10.3390/biomedicines9040375/s1, Figure S1. SDS-PAGE showing the result of the final purification step for SARS-CoV-2 3CLpro; Figure S2. Far-UV circular dichroism spectrum of the 3CLpro-rutin complex.

## References

[1] Cucinotta D., Vanelli M. (2020). WHO Declares COVID-19 a Pandemic. Acta BioMed..

[2] Painter E.M., Ussery E.N., Patel A., Hughes M.M., Zell E.R., Moulia D.L., Scharf L.G., Lynch M., Ritchey M.D., Toblin R.L. (2021). Demographic Characteristics of Persons Vaccinated During the First Month of the COVID-19 Vaccination Program—United States, December 14, 2020–January 14, 2021. MMWR. Morb. Mortal. Wkly. Rep..

[3] Han Y.J., Lee K.H., Yoon S., Nam S.W., Ryu S., Seong D., Kim J.S., Lee J.Y., Yang J.W., Lee J.H. (2021). Treatment of severe acute respiratory syndrome (SARS), Middle East respiratory syndrome (MERS), and coronavirus disease 2019 (COVID-19): A systematic review of in vitro, in vivo, and clinical trials. Theranostics.

[4] Peng Y., Tao H., Satyanarayanan S.K., Jin K., Su H. (2021). A Comprehensive Summary of the Knowledge on COVID-19 Treatment. Aging Dis..

[5] Muhseen Z., Hameed A., Al-Hasani H., Ahmad S., Li G. (2021). Computational Determination of Potential Multiprotein Targeting Natural Compounds for Rational Drug Design Against SARS-COV-2. Molecules.

[6] Romeo I., Mesiti F., Lupia A., Alcaro S. (2021). Current Updates on Naturally Occurring Compounds Recognizing SARS-CoV-2 Druggable Targets. Molecules.

[7] Bhattacharya R., Dev K., Sourirajan A. (2021). Antiviral activity of bioactive phytocompounds against coronavirus: An update. J. Virol. Methods.

[8] Vougogiannopoulou K., Corona A., Tramontano E., Alexis M., Skaltsounis A.-L. (2021). Natural and Nature-Derived Products Targeting Human Coronaviruses. Molecules.

[9] Latos-Brozio M., Masek A. (2019). Structure-Activity Relationships Analysis of Monomeric and Polymeric Polyphenols (Quercetin, Rutin and Catechin) Obtained by Various Polymerization Methods. Chem. Biodivers..

[10] Magar R.T., Sohng J.K. (2020). A Review on Structure, Modifications and Structure-Activity Relation of Quercetin and Its Derivatives. J. Microbiol. Biotechnol..

[11] Gullón B., Lú-Chau T.A., Moreira M.T., Lema J.M., Eibes G. (2017). Rutin: A review on extraction, identification and purification methods, biological activities and approaches to enhance its bioavailability. Trends Food Sci. Technol..

[12] Lide D.R., Milne G.W.A. (1994). Handbook of data on organic compounds.

[13] Srinivas K., King J.W., Howard L.R., Monrad J.K. (2010). Solubility and solution thermodynamic properties of quercetin and quercetin dihydrate in subcritical water. J. Food Eng..

[14] Abian O., Ortega-Alarcon D., Jimenez-Alesanco A., Ceballos-Laita L., Vega S., Reyburn H.T., Rizzuti B., Velazquez-Campoy A. (2020). Structural stability of SARS-CoV-2 3CLpro and identification of quercetin as an inhibitor by experimental screening. Int. J. Biol. Macromol..

[15] Abian O., Vega S., Sancho J., Velázquez-Campoy A. (2013). Allosteric Inhibitors of the NS3 Protease from the Hepatitis C Virus. PLoS ONE.

[16] Hidalgo J., Latorre P., Carrodeguas J.A., Velázquez-Campoy A., Sancho J., Lopez-Buesa P. (2016). Inhibition of Pig Phosphoenolpyruvate Carboxykinase Isoenzymes by 3-Mercaptopicolinic Acid and Novel Inhibitors. PLoS ONE.

[17] Neira J.L., Bintz J., Arruebo M., Rizzuti B., Bonacci T., Vega S., Lanas A., Velázquez-Campoy A., Iovanna J.L., Abián O. (2017). Identification of a Drug Targeting an Intrinsically Disordered Protein Involved in Pancreatic Adenocarcinoma. Sci. Rep..

[18] Villanueva R., Romero-Tamayo S., Laplaza R., Martínez-Olivan J., Velázquez-Campoy A., Sancho J., Ferreira P., Medina M. (2019). Redox- and Ligand Binding-Dependent Conformational Ensembles in the Human Apoptosis-Inducing Factor Regulate Its Pro-Life and Cell Death Functions. Antioxid. Redox Signal..

[19] González A., Salillas S., Velázquez-Campoy A., Angarica V.E., Fillat M.F., Sancho J., Lanas A. (2019). Identifying potential novel drugs against Helicobacter pylori by targeting the essential response regulator HsrA. Sci. Rep..

[20] Santofimia-Castaño P., Xia Y., Lan W., Zhou Z., Huang C., Peng L., Soubeyran P., Velázquez-Campoy A., Abián O., Rizzuti B. (2019). Ligand-based design identifies a potent NUPR1 inhibitor exerting anticancer activity via necroptosis. J. Clin. Investig..

[21] Savov V.M., Galabov A.S., Tantcheva L.P., Mileva M.M., Pavlova E.L., Stoeva E.S., Braykova A.A. (2006). Effects of rutin and quercetin on monooxygenase activities in experimental influenza virus infection. Exp. Toxicol. Pathol..

[22] Jo S., Kim H., Kim S., Shin D.H., Kim M. (2019). Characteristics of flavonoids as potent MERS-CoV 3C-like protease inhibitors. Chem. Biol. Drug Des..

[23] Chen Y.K., Chen S.Q., Li X., Zeng S. (2005). Quantitative regioselectivity of glucuronidation of quercetin by recombinant UDP-glucuronosyltransferases 1A9 and 1A3 using enzymatic kinetic parameters. Xenobiotica.

[24] Matsumoto M., Matsukawa N., Mineo H., Chiji H., Hara H. (2004). A Soluble Flavonoid-glycoside, αG-Rutin, Is Absorbed as Glycosides in the Isolated Gastric and Intestinal Mucosa. Biosci. Biotechnol. Biochem..

[25] Pedriali C.A., Fernandes A.U., Bernusso L.D.C., Polakiewicz B. (2008). The synthesis of a water-soluble derivative of rutin as an antiradical agent. Quim. Nova.

[26] Park K.H., Choi J.M., Cho E., Jeong D., Shinde V.V., Kim H., Choi Y., Jung S. (2017). Enhancement of Solubility and Bioavailability of Quercetin by Inclusion Complexation with the Cavity of Mono-6-deoxy-6-aminoethylamino-β-cyclodextrin. Bull. Korean Chem. Soc..

[27] Iacopetta D., Grande F., Caruso A., Mordocco R.A., Plutino M.R., Scrivano L., Ceramella J., Muià N., Saturnino C., Puoci F. (2017). New insights for the use of quercetin analogs in cancer treatment. Future Med. Chem..

[28] Nettore I.C., Rocca C., Mancino G., Albano L., Amelio D., Grande F., Puoci F., Pasqua T., Desiderio S., Mazza R. (2019). Quercetin and its derivative Q2 modulate chromatin dynamics in adipogenesis and Q2 prevents obesity and metabolic disorders in rats. J. Nutr. Biochem..

[29] Lee H., Mittal A., Patel K., Gatuz J.L., Truong L., Torres J., Mulhearn D.C., Johnson M.E. (2014). Identification of novel drug scaffolds for inhibition of SARS-CoV 3-Chymotrypsin-like protease using virtual and high-throughput screenings. Bioorganic Med. Chem..

[30] Zhang L., Lin D., Sun X., Curth U., Drosten C., Sauerhering L., Becker S., Rox K., Hilgenfeld R. (2020). Crystal structure of SARS-CoV-2 main protease provides a basis for design of improved α-ketoamide inhibitors. Science.

[31] Trott O., Olson A.J. (2010). AutoDock Vina: Improving the speed and accuracy of docking with a new scoring function, efficient optimization, and multithreading. J. Comput. Chem..

[32] Morris G.M., Goodsell D.S., Halliday R.S., Huey R., Hart W.E., Belew R.K., Olson A.J. (1998). Automated docking using a La-marckian genetic algorithm and an empirical binding free energy function. J. Comput. Chem..

[33] Komoto J., Yamada T., Watanabe K., Takusagawa F. (2004). Crystal Structure of Human Prostaglandin F Synthase (AKR1C3). Biochemistry.

[34] Pettersen E.F., Goddard T.D., Huang C.C., Couch G.S., Greenblatt D.M., Meng E.C., Ferrin T.E. (2004). UCSF Chimera—A visualization system for exploratory research and analysis. J. Comput. Chem..

[35] Santofimia-Castaño P., Rizzuti B., Pey Á.L., Soubeyran P., Vidal M., Urrutia R., Iovanna J.L., Neira J.L. (2017). Intrinsically disordered chromatin protein NUPR1 binds to the C-terminal region of Polycomb RING1B. Proc. Natl. Acad. Sci. USA.

[36] Abraham M.J., Murtola T., Schulz R., Páll S., Smith J.C., Hess B., Lindahl E. (2015). GROMACS: High performance molecular simulations through multi-level parallelism from laptops to supercomputers. SoftwareX.

[37] Lindorff-Larsen K., Piana S., Palmo K., Maragakis P., Klepeis J.L., Dror R.O., Shaw D.E. (2010). Improved side-chain torsion potentials for the Amber ff99SB protein force field. Proteins Struct. Funct. Bioinform..

[38] Wang J., Wolf R.M., Caldwell J.W., Kollman P.A., Case D.A. (2004). Development and testing of a general amber force field. J. Comput. Chem..

[39] Jorgensen W.L., Chandrasekhar J., Madura J.D., Impey R.W., Klein M.L. (1983). Comparison of simple potential functions for simulating liquid water. J. Chem. Phys..

[40] Guzzi R., Rizzuti B., Bartucci R. (2012). Dynamics and Binding Affinity of Spin-Labeled Stearic Acids in β-Lactoglobulin: Evidences from EPR Spectroscopy and Molecular Dynamics Simulation. J. Phys. Chem. B.

[41] Neira J.L., Rizzuti B., Iovanna J.L. (2016). Determinants of the pKa values of ionizable residues in an intrinsically disordered protein. Arch. Biochem. Biophys..

[42] Guglielmelli A., Rizzuti B., Guzzi R. (2018). Stereoselective and domain-specific effects of ibuprofen on the thermal stability of human serum albumin. Eur. J. Pharm. Sci..

[43] Bacha U., Barrila J., Velazquez-Campoy A., Leavitt S.A., Freire E. (2004). Identification of Novel Inhibitors of the SARS Coronavirus Main Protease 3CLpro. Biochemistry.

[44] Tian W., Chen C., Lei X., Zhao J., Liang J. (2018). CASTp 3.0: Computed atlas of surface topography of proteins. Nucleic Acids Res..

[45] Rizzuti B., Grande F. (2020). Virtual screening in drug discovery: A precious tool for a still-demanding challenge. Protein Homeostasis Diseases.

[46] Komatsu T.S., Okimoto N., Koyama Y.M., Hirano Y., Morimoto G., Ohno Y., Taiji M. (2020). Drug binding dynamics of the dimeric SARS-CoV-2 main protease, determined by molecular dynamics simulation. Sci. Rep..

[47] Suárez D., Díaz N. (2020). SARS-CoV-2 Main Protease: A Molecular Dynamics Study. J. Chem. Inf. Model..

[48] Martinez C.R., Iverson B.L. (2012). Rethinking the term “pi-stacking”. Chem. Sci..

[49] Avasthi K., Shukla L., Kant R., Ravikumar K. (2014). Folded conformations due to arene interactions in dissymmetric and symmetric butylidene-linker models based on pyrazolo[3,4-d]pyrimidine, purine and 7-deazapurine. Acta Crystallogr. Sect. C Struct. Chem..

[50] Ghahremanpour M.M., Tirado-Rives J., Deshmukh M., Ippolito J.A., Zhang C.-H., De Vaca I.C., Liosi M.-E., Anderson K.S., Jorgensen W.L. (2020). Identification of 14 Known Drugs as Inhibitors of the Main Protease of SARS-CoV-2. ACS Med. Chem. Lett..

[51] Chodera J., Lee A.A., London N., Von Delft F. (2020). Crowdsourcing drug discovery for pandemics. Nat. Chem..

[52] Chua L.S. (2013). A review on plant-based rutin extraction methods and its pharmacological activities. J. Ethnopharmacol..

[53] Matsuo M., Sasaki N., Saga K., Kaneko T. (2005). Cytotoxicity of Flavonoids toward Cultured Normal Human Cells. Biol. Pharm. Bull..

[54] Iriti M., Kubina R., Cochis A., Sorrentino R., Varoni E.M., Kabała-Dzik A., Azzimonti B., Dziedzic A., Rimondini L., Wojtyczka R.D. (2017). Rutin, a Quercetin Glycoside, Restores Chemosensitivity in Human Breast Cancer Cells. Phytother. Res..

[55] Al-Zahrani A.A. (2020). Rutin as a Promising Inhibitor of Main Protease and Other Protein Targets of COVID-19: In Silico Study. Nat. Prod. Commun..

[56] Puttaswamy H., Gowtham H.G., Ojha M.D., Yadav A., Choudhir G., Raguraman V., Kongkham B., Selvaraju K., Shareef S., Gehlot P. (2020). In silico studies evidenced the role of structurally diverse plant secondary metabolites in reducing SARS-CoV-2 pathogenesis. Sci. Rep..

[57] Xu Z., Yang L., Zhang X., Zhang Q., Yang Z., Liu Y., Wei S., Liu W. (2020). Discovery of Potential Flavonoid Inhibitors Against COVID-19 3CL Proteinase Based on Virtual Screening Strategy. Front. Mol. Biosci..

[58] Cherrak S.A., Merzouk H., Mokhtari-Soulimane N. (2020). Potential bioactive glycosylated flavonoids as SARS-CoV-2 main protease inhibitors: A molecular docking and simulation studies. PLoS ONE.

[59] Kumari A., Rajput V.S., Nagpal P., Kukrety H., Grover S., Grover A. (2020). Dual inhibition of SARS-CoV-2 spike and main protease through a repurposed drug, rutin. J. Biomol. Struct. Dyn..

[60] Huynh T., Wang H., Luan B. (2020). Structure-based lead optimization of herbal medicine rutin for inhibiting SARS-CoV-2’s main protease. Phys. Chem. Chem. Phys..

[61] Shivanika C., Kumar S.D., Ragunathan V., Tiwari P., Sumitha A., Devi P.B. (2020). Molecular docking, validation, dynamics simulations, and pharmacokinetic prediction of natural compounds against the SARS-CoV-2 main-protease. J. Biomol. Struct. Dyn..

[62] Verma S., Pandey A.K. (2021). Factual insights of the allosteric inhibition mechanism of SARS-CoV-2 main protease by quercetin: An in silico analysis. 3 Biotech.

[63] Nguyen T.T.H., Woo H.-J., Kang H.-K., Nguyen V.D., Kim Y.-M., Kim D.-W., Ahn S.-A., Xia Y., Kim D. (2012). Flavonoid-mediated inhibition of SARS coronavirus 3C-like protease expressed in Pichia pastoris. Biotechnol. Lett..

[64] Chen L., Li J., Luo C., Liu H., Xu W., Chen G., Liew O.W., Zhu W., Puah C.M., Shen X. (2006). Binding interaction of quercetin-3-β-galactoside and its synthetic derivatives with SARS-CoV 3CLpro: Structure–activity relationship studies reveal salient pharmacophore features. Bioorganic Med. Chem..

