# Supplementary material for: Rutin Is a Low Micromolar Inhibitor of SARS-CoV-2 Main Protease 3CLpro: Implications for Drug Design of Quercetin Analogs
Source: Biomedicines. 2021 Apr 2;9(4):375. doi: 10.3390/biomedicines9040375 (PMC8066963; doi:10.3390/biomedicines9040375)
Supplement: Supplementary file 1 [file biomedicines-09-00375-s001.pdf]

biomedicines

Supplementary Material

Rutin Is a Low Micromolar Inhibitor of SARS-CoV-2 Main Protease 3CLpro: Implications for Drug Design of Quercetin Analogs

Bruno Rizzuti [1,2*], Fedora Grande [3], Filomena Conforti [3], Ana Jimenez-Alesanco [2,4], Laura Ceballos-Laita [2,5], David Ortega-Alarcon [2,4], Sonia Vega [2], Hugh T. Reyburn [6], Olga Abian [2,4,5,7,8*] and Adrian Velazquez-Campoy [2,4,5,8,9*]

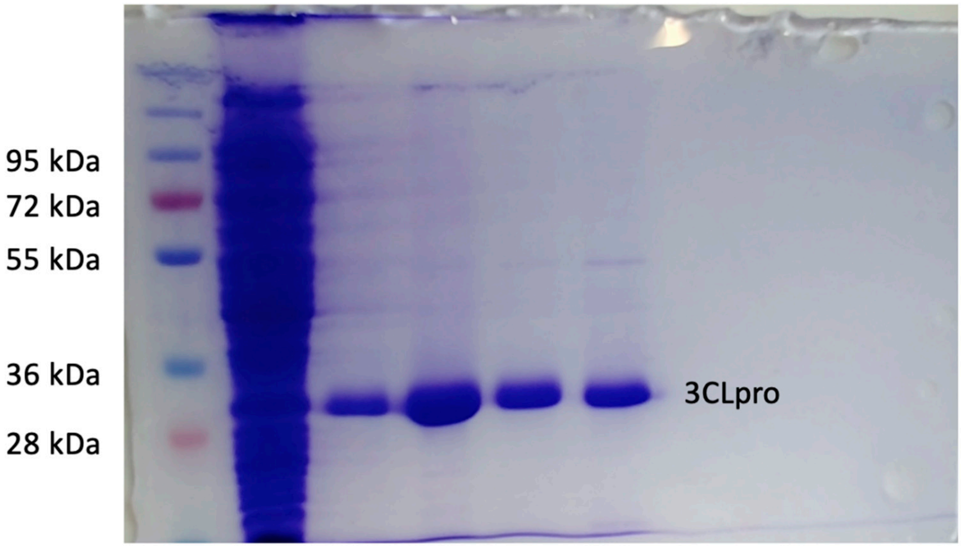

Figure S1. SDS-PAGE showing the result of the final purification step for SARS-CoV-2 3CLpro.

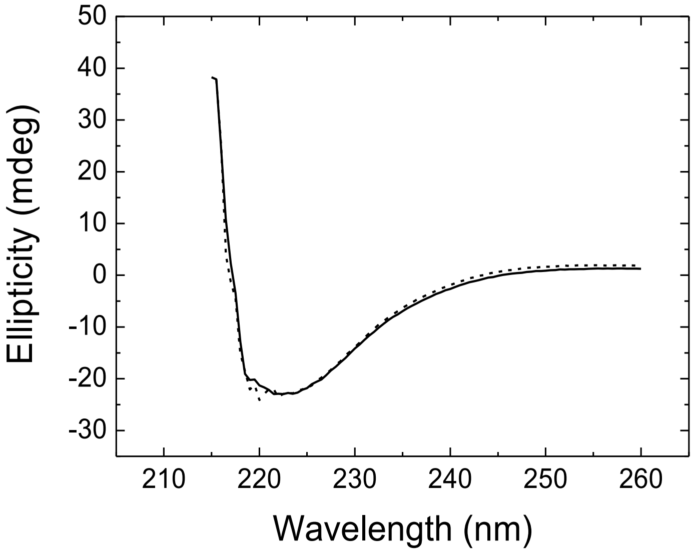

Figure S2. Far-UV circular dichroism spectrum of the 3CLpro-rutin complex (continuous line) and addition of individual spectra of 3CLpro and rutin (dashed line), recorded at 10 μM protein concentration and 100 μM ligand concentration, under the same conditions. The presence of DMSO at low concentration caused degradation of the signal below 215 nm, as reported previously (Abian et al. Int. J. Biol. Macromol. **2020**, *164*, 1693–1703).
